# What have we learned from observational studies and clinical trials of mild to moderate COPD?

**DOI:** 10.1186/s12931-018-0882-0

**Published:** 2018-09-17

**Authors:** Miriam Barrecheguren, Cruz González, Marc Miravitlles

**Affiliations:** 1 Pneumology Department, Hospital General Universitari Vall d’Hebron, CIBER de Enfermedades Respiratorias (CIBERES), P. de la Vall d’Hebron, 119-129, 08035 Barcelona, Spain; 2 Pneumology Department, Hospital Clínic Universitari, Valencia, Spain

**Keywords:** COPD, Diagnosis, Epidemiology, Guidelines, Mild-moderate disease, Physical activity, Quality of life, Treatment

## Abstract

Chronic obstructive pulmonary disease (COPD) is a major cause of morbidity and mortality worldwide. It is well established that patients with mild to moderate disease represent the majority of patients with COPD, and patients with mild COPD already have measurable physiological impairment with increased morbidity and a higher risk of mortality compared with healthy non-smoking individuals. However, this subpopulation is both underdiagnosed and undertreated. In addition, most clinical trials include cohorts of patients with worse lung function and quality of life, which are very different from the milder patients usually seen in primary care.

Clinical trials have shown that mild-moderate COPD patients present an improvement in lung function after treatment with long-acting bronchodilators (LABD). Inhaled therapy has also shown benefits in terms of symptoms, health-related quality of life (HRQL) and exacerbation prevention in this population. Early intervention might have also a positive effect to prevent functional impairment. Nevertheless, there is scarce evidence from randomised clinical trials and real-life studies about the importance of pharmacological treatment in early stages of COPD to improve long-term outcomes. New concepts such as clinically important deterioration may help to investigate the impact of interventions on the natural history of the disease.

## Background

Chronic obstructive pulmonary disease (COPD) is characterised by chronic airflow limitation, which is usually progressive. This disease is a major cause of morbidity and is currently the fourth leading cause of death worldwide [1, 2]. Although it is mostly related to exposure to tobacco smoking, patients exposed to biomass and pollution are also at risk of developing COPD.

The impact of COPD on each patient depends on the degree of airflow limitation, the severity of symptoms and comorbidities. The Global Initiative for Chronic Obstructive Lung Disease (GOLD) staging system categorises COPD into 4 severity stages (based on post-bronchodilator FEV1): stage I or mild, FEV1 ≥ 80% predicted; stage II or moderate, FEV1 ≥ 50% and < 80% predicted; stage III or severe, FEV1 ≥ 30% and < 50% predicted; and stage IV or very severe, FEV1 < 30% predicted [3]. As the disease progresses, there is greater restriction in daily activity with impaired quality of life and an increase of symptoms and exacerbations.

Nonetheless, the reduction in daily activity is already present in mild disease [4]. Since COPD is progressive, it is important to identify and treat patients at early stages in order to prevent further deterioration. Patients with mild (stage I) COPD already have measurable physiological impairment with increased morbidity and a higher risk of mortality compared with healthy non-smoking controls [5]. It is well established that patients with mild to moderate disease represent the majority of patients with COPD [6]. However, this subpopulation is both underdiagnosed and undertreated [7]. In addition, most clinical trials include cohorts of patients with worse lung function and worse quality of life, which are very different from those usually seen in primary care (PC) [8].

Encouragement of smoking cessation, in conjunction with management of symptoms and treating activity limitations and exacerbations by appropriate pharmacologic and non-pharmacologic management at the earliest possible stage can positively affect the impact and progression of the disease [9]. In recent years, newer strategies and a growing number of new pharmacologic agents to treat COPD have also been introduced and show promise in improving the management of COPD [10]. New disease lung models to predict patient-specific drug deposition are also being developed in order to individualise therapy [11].

The aim of this article is to review the current evidence on the epidemiology, natural history and management of mild to moderate COPD (defined as an FEV1 > 50% predicted), highlighting the importance of treatment in earlier stages of the disease to try to prevent progression.

## Epidemiology and natural history

The World Health Organization estimates that up to 328 million people around the world have COPD with a global prevalence of 1% [12]. This prevalence increases to 8–10% for individuals above 40 years old [13]. A recent systematic review aimed at estimating the prevalence of COPD across world regions among people aged 30 years or more found an overall prevalence of 10.7% in 1990, which increased to 11.7% in 2010 (14.3% in men and 7.6% in women) [14]. However, the prevalence of COPD varied widely among world regions, being the highest in the American region (14.1%), and the lowest in South East Asia (7.8%) [14]. This variability might be explained by differences in exposures and environmental and genetic factors. Indeed, according to the BOLD study, conducted at 12 sites around the world (*n* = 8775), the overall prevalence of COPD ranged from 11.4 to 26.1%; the prevalence of GOLD I being between 1.4–15.5% and the prevalence of GOLD II between 5.1 and 12.4% [6]. The Latin American Project for the Investigation of Obstructive Lung Disease (PLATINO) examined the prevalence of COPD in adults > 40 years of age and reported crude rates of GOLD stage I between 5.2 and 12.5% and GOLD II between 1.9 and 6.4% in five Latin American countries [15]. Another systematic review estimated a pooled prevalence for GOLD I of 9.8% (95% confidence interval (CI) 5.9–15.8) and of 5.5% for GOLD II (95%CI 3.3–9.0) [13].

In Europe, a systematic review by Atsou et al. [16] found a prevalence of COPD from 2.1 to 26.1% depending on the country, the age group and the methods used, the majority being GOLD I and II.

In Spain, the IBERPOC study conducted in 1997 showed a mean prevalence of COPD of 9.1% in subjects between 40 and 69 years of age [17]. Ten years later, the EPI-SCAN trial estimated a prevalence of 10.2% in subjects between 40 and 80 years of age [18]. A sub-analysis of these studies reported an increase of patients in milder stages over time, ranging from 38.3% for mild and 39.7% for moderate in the 1997 study to 85.6% for mild and 13.0% for moderate in the 2007 study [19]. Despite the wide variability of the results found in these population-based studies, the majority of the patients diagnosed had mild or moderate COPD, independently of the classification criteria used (Table 1).

**Table 1:** Prevalence of COPD GOLD I and II in some epidemiological studies of COPD

Study	Country	Prevalence (%)	
		GOLD I	GOLD II
Halbert et al. [13]	Worldwide	All 7.6 (6.0–9.5)	
		9.8 (5.9–15.8)	5.5 (3.3–9.0)
Buist AS, et al. (BOLD) [6]	Guangzhou	5.9%	7.6%
	Salzburg	16.3%	9.3%
	Cape town	6.5%	14.2%
	Reykjavik	9.7%	6.7%
	Krakow	14.4%	10.3%
	Manila	0.9%	11.3%
Menezes AM, et al. (PLATINO) [15]	Sao Paulo	10.1%	4.6%
	Santiago	11%	4.9%
	Mexico City	5.2%	1.9%
	Montevideo	12.5%	6.4%
	Caracas	6.4%	4.9%
Miravitlles M et al. (EPISCAN) [18]	Spain	All 10.2%	
		5.7%	3.9%

One of the main concerns in COPD is underdiagnosis, which is more marked in early stages of the disease. It has been estimated that 73–78% of patients with GOLD stages I-II COPD remain undiagnosed [18]. Llordés et al. carried out a study to quantify the rate of underdiagnosis and the accuracy of COPD diagnosis in PC [20]. The study included 1738 subjects over the age of 45 with a history of smoking and found a rate of underdiagnosis of 73%. Recently, the same group performed a case-finding study in smokers or former smokers older than 40 years and found a prevalence of COPD of 26.7%, with the majority of patients being in GOLD stages I and II (42.1 and 49.1%, respectively) [21]. Up to 29% of patients with underdiagnosed COPD are asymptomatic [22], which contributes to late detection. In addition, patients often under-recognize the significance of respiratory symptoms, or they may accept that their symptoms are caused by smoking and ignore them rather than consult. Another cause for underdiagnosis might also be a deficient active search for COPD by health care professionals [21]. Some of the diagnostic initiatives that have been tested to improve COPD diagnosis are early detection programs [23] and other tools such as the use of peak-flow meters or mini spirometers [24], and screening questionnaires [21, 25].

In contrast to underdiagnosis, the definition of COPD based on FEV1/FVC instead of lower limit of normality (LLN) might lead to the overdiagnosis of mild COPD in individuals older than 65 years old [26].

The natural history of COPD is usually described by the rate of decline in lung function. The rate of decline in FEV1 in GOLD stages I-II is faster than in patients with stages III-IV [27–29]. Recently, in a cohort of smokers and ex-smokers, the COPD Gene group showed that functional alteration of the small airways measured by computerized tomography was significantly associated with a decline of FEV1, particularly in mild to moderate stages of the disease, and this association was also evident even before the detection of obstruction in the spirometry [30].

Exacerbations have a negative impact on the natural history of COPD, and their presence has been associated with a faster decline of FEV1, in particular in GOLD stagesI and II [31], in which the effect of each exacerbation on rate of FEV1 decline has been estimated to be − 23 ml/year in GOLD 1 and − 10 ml/year in GOLD 2 for any exacerbation, increasing up to -87 ml/year and -20 ml/year for GOLD 1 and 2 respectively for each severe exacerbation. Frequent exacerbators also have a worse quality of life and an increased risk of death [32–34]. Thus, the prevention of exacerbations should be a target for every COPD patient, irrespective of the severity of the disease.

## Symptoms, quality of life and physical activity

COPD is defined by the presence of chronic respiratory symptoms, among which exertional dyspnoea, cough and sputum production are the most frequent [35]. Although more severe patients present a higher intensity of symptoms, there is not always a clear relationship between the degree of airflow obstruction and the presence and impact of respiratory symptoms in a patient with COPD [35, 36]. The observational study ASSESS characterised the symptoms of COPD during 24 h and assessed their relationship with patient-reported outcomes [37]. The analysis showed that the frequency of symptom occurrence was high for any degree of bronchial obstruction, with a prevalence of symptoms in GOLD stages I-II of 84–88%.

The presence of respiratory symptoms in patients with early COPD may modify long-term FEV1 decline and health-related quality of life. Symptomatic subjects with GOLD I COPD have a more rapid long-term decline in lung function than asymptomatic subjects [38, 39].

Despite referring fewer symptoms, mild COPD patients present exercise limitation that leads to changes in lifestyle and deconditioning, with an increasing burden of the disease beyond the degree of airflow obstruction. Indeed, around two thirds of GOLD stage II patients may have significant lung hyperinflation [40]. This hyperinflation is manifested as exertional dyspnoea, and furthermore, dyspnoea significantly contributes to a worse quality of life due to the high impact on their daily activities [41]. Even mild patients may have severe dyspnoea; a study carried out in 5299 subjects found that 7.5 and 18.3% of subjects with GOLD stages I and II, respectively, had a Medical Research Council (MRC) score ≥ 3 [42]. Similarly, a large European observational study conducted in 1817 patients in PC showed a high prevalence of symptoms in GOLD II patients (cough 76.6%, sputum 64.1%, and dyspnoea 67.3%). Moreover, 31% had a MRC dyspnoea grade ≥ 3. [43]. In a recent analysis carried out in 49,438 COPD patients, 50.2% were GOLD stage II, and 37.1% had a MRC dyspnoea grade ≥ 3, demonstrating that significant dyspnoea can be present in early stages of the disease [44]. In addition to being the most frequent symptom, dyspnoea has proven to be a better predictor of death at 5 years than airway obstruction in patients with COPD [45].

Concerning quality of life, the study by Jones et al. [43] using the St. George’s Respiratory Questionnaire [SGRQ] and the 12-Item Short Form Survey [SF-12] demonstrated that GOLD I and II patients had an impaired quality of life compared with healthy individuals. These results are similar to those obtained in the EPI-SCAN study, which observed a significant impairment of health-related quality of life in all severities of COPD, even GOLD I and II and undiagnosed COPD patients [18].

Physical activity significantly deteriorates in patients with COPD as the disease progresses [46], and the reduction in physical activity is one of the best predictors for COPD survival [47]. In addition, physical inactivity is strongly associated with the presence of comorbidities [48]. In contrast, regular physical activity reduces hospitalisation and the risk of death in COPD [49]. However, even in early stages of the disease, most patients with COPD spend less time walking (and walking more slowly) and standing, and more time sitting and lying compared with sedentary healthy elderly subjects [50].

## Treatment of mild to moderate COPD

The main objectives for the treatment of COPD are the reduction of symptoms, the reduction of frequency and severity of the exacerbations and the improvement of prognosis [3, 51]. Non-pharmacological treatment, especially smoking cessation, is essential for every COPD patient and ha sproven to be the most efficient and cost-effective therapeutic strategy [3, 51]. Smoking cessation slows the loss of lung function and improves survival. Indeed, the benefits may be more evident in milder patients than in individuals with a more established disease [52]. Psychotherapy might be beneficial in addition to exercise and medication. Other non-pharmacological approaches include regular physical activity, an adequate nutrition status, management of comorbidities, and vaccination against pneumococcal and influenza viruses is also recommended for all patients. Pulmonary rehabilitation has shown to improve symptoms, exercise tolerance and health-related quality of life, and to reduce the risk of morbidity from acute exacerbations of COPD [53]. Both a meta-analysis and a systematic review concluded that patients with mild to moderate COPD also benefit from short- and long-term rehabilitation [54, 55]. Hence, considering the benefits of physical activity, patients with COPD should be encouraged to increase their level of exercise from early stages.

### Real-life experience of treatment of mild-moderate COPD

Mild COPD patients are frequently followed in a PC setting, and they are usually not included in randomised controlled trials (RCT) [8], therefore, database studies could provide interesting data on the management of these subjects. An analysis of real-life prescribing patterns in primary care in the UK described the treatment of 24,957 COPD patients among which half were GOLD II [56]. Up to 17.7% did not receive any treatment for their disease, and 14% were treated only with short-acting β-agonists (SABA) despite being symptomatic. In fact, two thirds of the untreated patients referred a COPD Assessment Test (CAT) score > 10, while 22.7% had a modified MRC ≥ 2, consistent with highly symptomatic disease. Moreover, a high percentage of treated patients remained symptomatic: 36.6% of patients had a modified MRC ≥ 2, and 76.4% had a CAT score ≥ 10 with their current treatment regimen.

In a similar study that included 7881 newly diagnosed COPD patients in PC, 54.3% had GOLD stage II at the time of diagnosis. Initially, 37.9% were treated only with short-acting bronchodilators (SABD) or did not receive any inhaled treatment, while only one third of the patients were prescribed a long-acting bronchodilator (LABD), alone or in combination with inhaled corticosteroids (ICS) [57]. Raluy-Callado et al. [58] described the treatment patterns in a population of prevalent and incident COPD patients, of whom 53.5 and 61% were GOLD stage II, respectively. The authors found that almost half of the incident patients with FEV1 > 50% and infrequent exacerbations were prescribed monotherapy, being SABD the most frequent (29.3%) [58]. Similar results have been observed in other countries. In Catalonia (Spain), a recent study showed that 55.2% of the patients diagnosed in PC had GOLD stage II COPD. After the diagnosis of the disease, GOLD II patients frequently remained untreated (28.1%) or were treated only with a SABD (18.5%) [59].

The reasons for undertreatment are varied. COPD patients often do not seek medical help until the symptoms interfere with their daily life, and asymptomatic patients are also less likely to be identified and to receive treatment. In addition, some patients tend to reduce their physical activity even at early stages of the disease, which might help to minimize symptom perception. Moreover, mild COPD patients are more likely to experience unreported exacerbations [60], which may contribute to an underestimation of the impact of COPD. Another possible reason for undertreatment is a lack of awareness of guideline recommendations.

### Clinical trials of pharmacological treatment of mild to moderate COPD

Clinical trials usually include patients with moderate or severe COPD; hence, the data available on the benefits of inhaled treatment in mild COPD patients are limited. This is in contrast with the distribution of severity in a real-life setting, with up to 50% of the COPD patients controlled in PC being GOLD stage II [16, 59]. However, there is some evidence of the efficacy of treatments in GOLD stage II COPD patients derived from studies on mild-moderate COPD and subgroup analysis of RCTs (Table 2).

**Table 2:** Results in GOLD stage II COPD patients in randomised clinical trials

Study	Treatment	GOLD stage II patients (n)	Results
Decramer M, et al. UPLIFT study [66]	Tiotropium vs. placebo	2739	- Lower rate of decline of mean postbronchodilator FEV1 - improvement in the SGRQ - increase in the time to first exacerbation
Beeh KM, et al. [61]	Tiotropium vs. placebo	586	- increment in trough FEV1
Duser D, et al. Mistral study [68]	Tiotropium vs. placebo	426	- reduction in the number of exacerbations
Freeman D, et al. SPRUCE study [62]	Tiotropium	185	- improvement in trough FEV1 - reduction in the number of exacerbations
Singh D, et al. OTEMTO study [64]	Tiotropium/olodaterol vs. tiotropium or placebo	1042	- improvement in the trough FEV1 - improvement in symptoms and the SGRQ
Vincken W, et al. [88]	Tiotropium vs. ipratropium	535	- improvement in trough FEV1 - improvement in PEFR, salbutamol use, TDI, and SGRQ - reduction in the number of exacerbations
Casburi R, et al. [89]	Tiotropium vs. placebo	921	- improvement in trough FEV1 - reduction in dyspnoea - improvement in health status scores - reduction in the number of exacerbations
Jenkins C, et al. TORCH study [63]	SFC vs. placebo	2156	- reduction in the risk of death - improvements in FEV1 - reduction in the annual rate of exacerbations
Jones PW, et al. ISOLDE study [90]	Fluticasone propionate vs. placebo	391 (mild) 359 (moderate-severe)	- reduction in the number of exacerbations (less in mild disease)
Vestbo J, et al. SUMMIT study [77]	Fluticasone fuorate vs. placebo vs. vilanterol vs. combination therapy	4135 vs 4121 vs 4118	- reduction in the rate of decline in FEV1 - reduction in the rate of moderate and severe exacerbation
Vogelmeier CF, et al. CRYSTAL study [65]	Indacaterol/glycopyrronium vs. LABA/ICS vs. LABA or LAMA	811 vs. 269 811 vs. 268	- greater improvement on trough FEV1 - greater improvement in dyspnoea - greater improvement in health status - lower rescue medication use
Wedzicha JA, et al. [69]	Indacaterol/glycopyrronium vs. SFC	1680 vs. 1682	- superiority in reducing annual rate of exacerbations - longer time to the first exacerbation

*FEV1* forced expiratory volume in one second, *ICS* inhaled corticosteroids, *LABA* long-acting β2-agonist, *LAMA* long-acting muscarinic antagonist, *PEFR* peak expiratory flow rate, *SFC* inhaled salmeterol plus fluticasone propionate, *SGRQ* St George’s Respiratory Questionnaire, *TDI* transition dyspnoea index

#### Lung function

The effects of the treatments are measured by different outcomes: lung function, dyspnoea, quality of life and exacerbations. In terms of lung function, in a 12-week placebo-controlled study conducted in Germany, treatment with tiotropium showed a significant increase of trough FEV1 (+ 79 ml), which was even more pronounced in COPD patients with a FEV1 between 50 and 70% (trough FEV1 + 113 ml) [61]. In a PC setting, GOLD I and II patients also showed a trend to improving trough FEV1 with tiotropium compared with placebo after 12 weeks [effect size 40 ml (95%CI -30, 100)] [62].

The efficacy of the inhaled salmeterol/fluticasone combination (SFC) in patients with mild COPD is limited. A post hoc analysis of the TORCH study, which included 2156 subjects with FEV1 > 50% showed an increase in FEV1 in GOLD II patients treated with 101 ml of SFC compared with placebo [63].

The combination of a long-acting antimuscarinic agent (LAMA) and a long-acting beta-2 agonist (LABA) (tiotropium/olodaterol) has also shown to improve trough FEV1 compared to placebo (149 ml versus − 10 ml, respectively) in GOLD stage II patients [64]. In the CRYSTAL study, which included 2160 patients with moderate COPD, the effect of the LABA/LAMA indacaterol/glycopyrronium was superior to LABA/ICS in trough FEV1 at week 12 (treatment difference + 71 ml) and to LABA or LAMA monotherapies (difference + 101 ml) [65].

#### Dyspnoea

Inhaled therapy has also shown benefits in GOLD II patients in terms of symptoms. Treatment with tiotropium/olodaterol improved dyspnoea measured by the Transition Dyspnea Index (TDI) compared to placebo (1.67*versus* 0.39) [64]. The CRYSTAL study also showed superior improvement in the total TDI score with indacaterol/glycopyrronium versus LABA/ICS (difference 1.10 units) and versus LABA or LAMA (difference 1.26 units) in GOLD stage II COPD patients [65].

#### Quality of life

Regarding quality of life, in the UPLIFT study, the authors observed an improvement in health status measured by the SGRQ at all time points in the tiotropium group (*p* ≤ 0.006 for all time points) [66]. In the TORCH study, GOLD II COPD patients also experienced a better health status when treated with SFC in comparison with placebo (change in the SGRQ − 2.3), although the minimal clinically important difference was not reached [67]. The combination of tiotropium/olodaterol showed a greater improvement, with a change from baseline in the SGRQ score of − 4.7 compared with − 2.2 in the tiotropium arm, and − 0.7 in the placebo arm in GOLD II patients [64].

#### Exacerbations

The effects of treatment on exacerbation prevention in moderate COPD have been demonstrated in several studies. Tiotropium has shown to prevent exacerbations in patients with less severe COPD. In the MISTRAL study, the number of exacerbations in the subgroup of patients with a FEV1 ≥ 50% was lower for those receiving tiotropium compared to placebo (1.21 versus 1.97, respectively; *p* < 0.01) [68]. The UPLIFT study also showed that the time to the first exacerbation and the time to exacerbation resulting in hospital admission were longer in the tiotropium group than in the control group in the GOLD II subgroup (hazard ratio 0.82, 95% CI 0.75–0.90, and 0.74, 0.62–0.88, respectively) [66]. In PC, patients with FEV1 > 50% that received tiotropium in addition to their usual treatment were less likely to have ≥1 exacerbation during follow-up compared to the placebo group (6.8% versus 16.8%) [62].

Treatment with SFC has also demonstrated to reduce the annual rate of exacerbations by 31% in GOLD stage II COPD patients compared with placebo (mean of 0.57/year versus 0.82/year, respectively) [68]. The combination LABA/LAMA is also effective in reducing exacerbations. In fact, the superiority of indacaterol/glycopyrronium over monotherapies and LABA/ICS was demonstrated in several phase III clinical trials, including the FLAME study, which showed that this combination was superior to SFC in reducing the annual rate of exacerbations, although in patients with moderate COPD the difference did not reach significance, probably due to an insufficient sample size of patients with this level of severity (HR 0.93 (0.82–1.06)) [69].

These studies suggest that in general, treatment of mild to moderate COPD should be started with one or a combination of two LABD. The only exception would be asthma-COPD overlap (ACO) patients, who usually have more respiratory symptoms, but especially a better response to ICS [70].

## Impact of early treatment on the natural history of COPD

Since the decline in lung function is faster in early stages of the disease, early intervention is required to prevent functional impairment [71]. However, there are only a few large-scale clinical trials on long-term interventions in patients in early stages of COPD. The Lung Health Study, carried out in 3926 smokers with mild to moderate airway obstruction for a period of 5 years showed that participants who stopped smoking experienced an improvement in FEV1 in the year after quitting (average of 47 ml or 2%), and that the subsequent rate of decline in FEV1 among sustained quitters was half the rate of that of continuing smokers (31 ml versus 62 ml, respectively), and comparable to that of never-smokers [72]. A post hoc analysis of the UPLIFT study that included only GOLD II patients observed that the rate of decline for postbronchodilator FEV1 was lower in the tiotropium group than in the control group (43 ml per year [SE 2] vs. 49 ml per year [SE 2], *p* = 0.024) [66]. Another sub-analysis from UPLIFT assessed the efficacy of treatment with tiotropium in patients without previous maintenance treatment (naïve patients) and included a majority of GOLD II (60%). The annual FEV1 decline was slower in the tiotropium group (35 ml (SD 3) in the tioptropium arm vs. 45 ml (SD 4) in the placebo arm) [73].

More recently, in a randomised placebo-controlled trial which included 841 mild to moderate COPD patients, Zhou et al. [74] observed that the annual FEV1 decline in early stage COPD was lower in patients treated with tiotropium (29 ml ±5) than in the placebo group (51 ml ± 6; difference 22 ml (95% CI 6 to 37). In the TORCH study, SFC also showed a reduction in the rate of decline in FEV1 versus placebo in the GOLD II group (difference 16 ml/year, 95% CI: 0, 32) [63]. These results were confirmed in the SUMMIT study, in which GOLD II patients also had a lower rate of decline in FEV1 in the fluticasone furoate/vilanterol arm compared to placebo (38 ml (2.4) vs. -46 ml (2.5) respectively, *p* = 0.019) [75]. These results should be considered in the context of both studies failing to reach their primary outcome of mortality (Table 3).

**Table 3:** Impact of early pharmacological treatment on rate of FEV1 decline

Study	Participants	Follow-up	Intervention	Outcome
Scanlon PD et al. [72]	3926 smokers	5 years	Quitting smoking	Improvement in FEV1 (47 ml or 2%)
Decreamer M, et al. [66]	2375 GOLD II	4 years	Tiotropium vs. placebo	Reduction in rate of FEV1 decline (43 ml/year vs. 49 ml/year, *p* = 0.024)
Troosters T et al. [73]	120 GOLD II	4 years	Tiotropium vs. placebo	Reduction in rate of FEV1 decline (35 ml vs. 45 ml)
Zhou Y et al. [74]	841 GOLD I and II	2 years	Tiotropium vs. placebo	Reduction in rate of FEV1 decline (29 ml vs. 51 ml)
Jenkins CR et al. [63]	2156 GOLD II	3 years	SFC vs. placebo	Reduction in rate of FEV1 decline (difference 16 ml/year, 95% CI 0,32)
Calverley PMA, et al. [75]	6981 GOLD II	15 to 44 months	SFC vs. placebo	Reduction in rate of FEV1 decline (38 ml vs −46 ml, *p* = 0.019))

*FEV1* forced expiratory volume in one second, *SFC* salmeterol/fluticasone fixed dose combination

With regard to mortality, the TORCH study showed a 33% reduction in the risk of death in GOLD stage II COPD patients (HR 0.67; 95% CI: 0.45, 0.98) with SFC compared with placebo [63]. In UPLIFT, there was a non significant reduction in mortality with tiotropium compared to placebo (hazard ratio [HR], 0.89; 95% CI, 0.79 to 1.02, *p* = 0.09) [76]. More recently, treatment with fluticasone furoate/vilanterol showed no effect on mortality in COPD patients with FEV1 > 50% and cardiovascular risk (HR 0·88 [95% CI 0·74–1·04]; 12% relative reduction; *p* = 0.137) [77].

### Deterioration prevention

Improvement of clinical and/or spirometric parameters are the endpoints routinely assessed to evaluate the effects of treatments in COPD in most RCTs. However, many patients do not improve and suffer progressive deterioration of their disease. Taking into account that deterioration of lung function, exacerbation frequency, and health status are important parameters to reflect the impact of treatment on COPD management [32, 78–81], the evaluation of composite endpoints may be more sensitive than individual measures to the effects of therapeutic interventions. One of the proposed composite endpoints is the so-called clinically important deterioration (CID). This endpoint allows assessing the rate of deterioration of lung function, exacerbation rate and/or health status of the patient and evaluates the effects of treatment. In addition, this composite endpoint is consistent with the current GOLD strategy, which recommends that lung function, COPD exacerbation risk, and health status are considered when assessing disease progression and severity [3].

A post hoc pooled analysis of three RCTs assessed short-term CID in maintenance-naïve patients with COPD receiving the combination of umeclidinium and vilanterol(LAMA/LABA) compared with tiotropium for 6 months [82].The results showed that early use of dual-bronchodilator therapy has superior efficacy in lung function and may reduce the risk of short-term CID compared to monotherapy in symptomatic patients with COPD [82].

Another analysis described the effect of the combination of another LAMA/LABA, indacaterol/glycopirronium versus both tiotropium or SFC on the prevention of CID using patient data from three large phase 3 RCTs [83]. The study used two different definitions for CID: definition 1 was a composite of ≥100 ml decrease in FEV1, a ≥ 4-unit increase in SGRQ, and a moderate to severe COPD exacerbation, while for definition 2, the FEV1 component was replaced by a ≥ 1-unit decrease in the TDI. This analysis confirmed the utility of the CID endpoint as a measurement of COPD worsening in patients with moderate to severe COPD and using this endpoint the analysis showed that the combination of indacaterol/glycopyrronium offers significant benefits over treatment with a LAMA or a LABA/ICS both in terms of the incidence and time to CID.

Another post hoc analysis evaluated CID using data from two randomised phase III studies that assessed the efficacy and safety of the combination of aclidinium/formoterol in patients with COPD [84]. The results demonstrated that this combination reduced the risk of a first CID, providing greater airway stability, and therefore, fewer deteriorations in lung function, dyspnoea, risk of exacerbations, and health status compared with placebo or monotherapies.

## Current treatment guidelines

A recent review of COPD treatment guidelines published in Europe and Russia in the last 7 years found that although there were differences in some recommended treatments, there was a general agreement on treatment goals and the use of LABD as the cornerstone of COPD treatment [85].

The GOLD strategy establishes pharmacologic treatment algorithms by groups of different risk and intensity of symptoms. For patients in GOLD group A (low risk and low symptom burden), a bronchodilator should be offered to reduce breathlessness. For patients in GOLD group B (low risk and high symptom burden), the preferred initial therapy should be a LABD. If the symptoms persist, therapy with two bronchodilators may be considered [3].

The National Institute for Health and Care Excellence (NICE) guidelines updated in 2010 recommended that patients with FEV1 > 50% predicted with exacerbations or persistent airflow obstruction should be offered LABDs, and in the case of persistence of symptoms, patients should receive either LABA/ICS or LAMA/LABA [86].

The Spanish COPD guidelines (GesEPOC) were developed based on risk stratification and clinical phenotypes [51]. They use an easy risk classification (low or high risk) based on lung function, dyspnoea grade, and history of exacerbations. The four clinical phenotypes identified were: non-exacerbator, ACO, exacerbator with emphysema, and exacerbator with chronic bronchitis. However, they only recommend the determination of clinical phenotype in high-risk patients. Pharmacological recommendations for low-risk patients consist of LABD without any type of anti-inflammatory treatment, and the initial treatment for most high risk patients consists of LAMA/LABA [51].

**Fig. 1:**
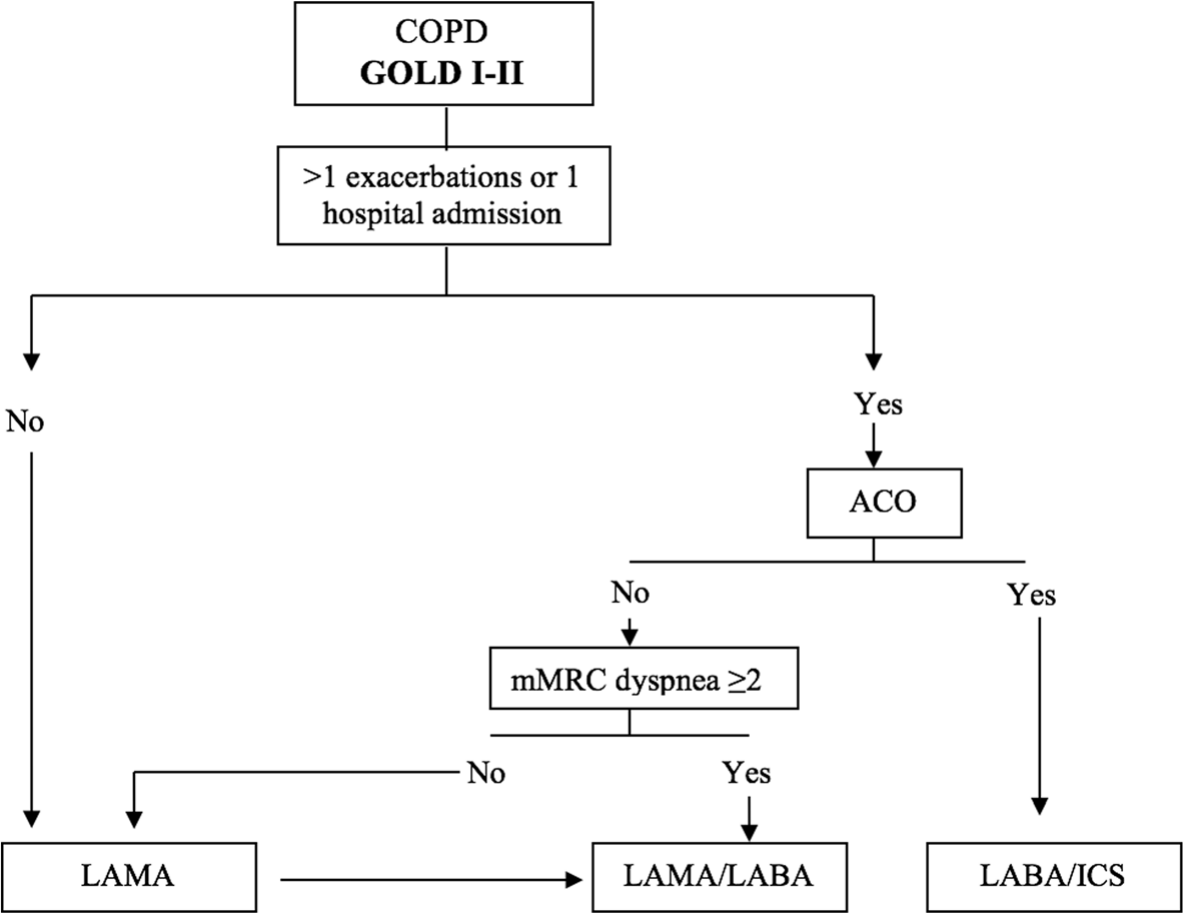
Proposal of treatment algorithm for the management of GOLD 1–2 COPD patients. COPD: chronic obstructive pulmonary disease; ACO: asthma-COPD overlap; LAMA: long acting anti muscarinic; LABA: long acting b-2 agonists; ICS: inhaled corticosteroids

The newest position statement of the Canadian Thoracic Society in COPD gives more weight to symptoms and exacerbations when it comes to increasing or decreasing therapy. In symptomatic patients with stable COPD without frequent exacerbations, treatment should be started with a LABD, and if experiencing persistent or increased dyspnoea, exercise intolerance, and/or reduced health status despite the use of monotherapy, patients should be considered for “step up” treatment with a LAMA/LABA. In patients with stable COPD experiencing exacerbations despite the use of LAMA or LABA monotherapy, “step up” treatment with inhaled LAMA/LABA should be considered. If a patient is still experiencing exacerbations despite the use of LAMA/LABA, “step up” treatment with LAMA plus LABA/ICS can be considered. Since inhaled triple or dual therapy may not prove to be superior in every patient, the notion of “step down” treatment may be a consideration in some patients. For patients with suspected ACO, a combination of LABA/ICS is recommended [87]. Figure 1 presents a treatment algorithm for mild-moderate COPD patients.

## Conclusions

Although patients with mild to moderate disease represent the majority of patients with COPD, most studies and in particular RCTs are focused on severe COPD patients. Moreover, mild to moderate patients tend to be underdiagnosed and undertreated, affecting their prognosis and quality of life. Since COPD is progressive, it is important to identify and treat patients at early stages in order to prevent further deterioration, even more considering that patients with mild COPD already have measurable physiological impairment that leads to changes in lifestyle and deconditioning.

Smoking cessation, management of symptoms and treating activity limitations and exacerbations by appropriate pharmacologic and non-pharmacologic management at the earliest possible stage could positively affect the impact and progression of the disease, symptoms, exacerbations, and quality of life. These benefits may be more evident in milder patients than in COPD individuals with a more established disease. Non-pharmacological management includes regular physical activity, pulmonary rehabilitation, an adequate nutrition status, and management of comorbidities, and vaccination against pneumococcal and influenza viruses is also recommended for all patients. On the other hand, early treatment with one LABD or the combination of two bronchodilators has shown to produce a slightly greater improvement in lung function in patients with GOLD stage II than in those with more advanced stages of COPD, and this improvement was associated with an improvement of symptoms, health-related quality of life and a reduction in the risk of exacerbations.

New studies suggest that early treatment with dual bronchodilators may prevent disease deterioration, but this interesting concept should be tested in large clinical trials specifically designed for this objective.

## Abbreviations

CAT: COPD Assessment Test
CID: Clinically important deterioration
COPD: Chronic obstructive pulmonary disease
FEV1: Forced expiratory volume in the first second
GOLD: Global Initiative for Chronic Obstructive Lung Disease
ICS: Inhaled corticosteroids
LABA: Long-acting β-2 agonist
LABD: Long-acting bronchodilator
LAMA: Long-acting antimuscarinic agent
MRC: Medical Research Council
PC: Primary care
RCT: Randomised clinical trial
SABA: Short-acting β-agonists
SABD: Short-acting bronchodilators
SF-12: 12-item short form survey
SFC: Salmeterol/fluticasone combination
SGRQ: St. George’s Respiratory Questionnaire
TDI: Transition dyspnoea index
